# Not all dementias are equally created: Tailored translational research differentially addresses unanswered questions

**DOI:** 10.3389/frdem.2022.1043624

**Published:** 2022-10-14

**Authors:** Charbel Moussa

**Affiliations:** ^1^Georgetown University, Washington, DC, United States; ^2^Georgetown University Medical Center, Washington, DC, United States

**Keywords:** dementia, continuum, biomarker, Alzheimer, methodology

Advances in the diagnostics of various dementias, including Alzheimer's disease (AD), Dementia with Lewy Bodies (DLB), Parkinson's disease dementia (PDD), clinical and behavioral variants of Frontotemporal Lobar Degeneration (FLD) and Parkinsonism with dementia, i.e., corticobasal degeneration (CBD) etc. have led to some understanding of the distinct and perhaps disparate anatomical, molecular and pathological pathways of dementia. Anatomical and molecular imaging, longitudinal post-mortem specimen studies and bio fluids (plasma, cerebrospinal fluid, tear, saliva, skin biopsies etc.) analysis all contributed to a thematic understanding that divergent neuropathology may lead to differential diagnoses that are underscored by dementia as a common denominator. While various methodologies are important in elucidating relevant mechanisms of dementia, a translational approach that takes into account a specific patient population could provide more adequate frontiers to establish precision of diagnosis and greater effectiveness of dementia therapeutics. In this context, translational research is a two-way process that uses pre-clinical science, technology and advances in various methodologies to validate mechanisms of human disease in reference to observational and clinical data, including clinical practice and clinical trials, as well as biomarker analysis, to support an innovative approach to advance dementia-specific therapies.

Translational Research in Dementia publishes high-quality basic research and pre-clinical data that are translatable to human to generate treatments and disease management across many areas of dementia. Primarily, there is a dire need to better understand how dementias vary in the human population (e.g., age, gender, ethnicity, geographical locations, cultures norms and practices, genotype etc…) and in disease, so adequate research tools and animal models can be generated. There is also need for clinical testing that accurately identify the type of dementia and cognitive impairment on a spectrum from prodromal disease, mild cognitive impairment (MCI), executive dysfunction and activities of daily living to forgetfulness and complete disability. Defining the disease stage and dementia type are critical to applicability of tools of research and models of disease. Dementia is highly variable and it is a continuum of symptoms with specific underlying *anatomical and molecular degenerative processes that render dementia to multiple diseases* that can only be studied *via* elucidation of specific events that are related to the type of dementia being studied.

Understanding dementias *via* adequate behavioral, psychiatric and clinical testing will foster translational research to master biological disease mechanisms using animal models, computational and biomedical engineering methods, and latest methods and technologies e.g., whole genome sequencing, to solidify therapeutic strategies and augment the chances of successful drug development. For example, artificial intelligence has emerged as a promising tool to study neurological diseases, including AD and related dementias, and machine learning could provide unbiased methods to classify types of dementia and/or determine the likelihood of a given class of drugs or therapies to potentially alter disease mechanisms. Furthermore, animal models provide powerful tools to study molecular mechanisms of dementia but they provide limited usefulness in capturing the spectrum of dementia in human. Large longitudinal and observation studies may be more relevant to understanding the symptomatic and behavioral development of dementia to elucidate its variabilities in the human population and develop platforms (tests, diagnostics, biomarkers etc.) to identify research tools that detect specific symptoms across the dementia continuum; and model symptoms in transgenic and other animal and computational models with specific biomarkers of disease. Tailoring a biomarker system that reliably measures dementia and its underlying molecular pathways heavily depends on disease stage, dementia type, scales and time point of therapeutic intervention with attention to drug actions either to manage symptoms or provide robust long-term disease-modifying effects. It is critically important that translational research leads to establishment of new therapies and initial determination of adequate pharmacokinetics and pharmacodynamics relationships in animal models and human subjects *via* rigorous pharmacology and demonstration of target engagement.

Translational research should increase our understanding of dementia and bridge the gap between pre-clinical evidence of disease mechanisms and therapeutic strategies to modify disease processes in humans *via* pharmacological (i.e., small molecules, biologics, gene and stem cell therapy etc…) and non-pharmacological (behavioral, music, exercise, dancing…) interventions. Translational research must provide adequate tools, methods, and research methodologies to overcome the “Valley of Death” and galvanize our ability to translate pre-clinical and observational findings into knowledge of disease mechanisms to measure and interpret data in a specific disease context. Reserve-translation could involve the use of human genomic, epigenomics and epigenetics data, proteomics and other omics to understand longitudinal and cross sectional changes in patients in order to develop testing and validation platforms of disease mechanisms and potential drug targets, respectively.

Translational Research in Dementia targets a diverse and multidisciplinary audience, including scientists, physicians and other clinicians, patients and patient advocacy groups and policy makers. For example, a U.S. congressional mission to find a treatment for AD by year 2025 is an important task that highlights the profound impact of dementia on U.S. society and globally. Translational Research in Dementia provides a global open access platform for researchers, in academia and industry, physicians and educators to better understand the limitations and strengths of the proposed hypotheses and mechanisms of actions, design, systems and methods to measure the impacts of interventions in preclinical research and provide feasibility for clinical studies and non-pharmacological interventions to treat dementia.

## Author contributions

The author confirms being the sole contributor of this work and has approved it for publication.

## Conflict of interest

CM is listed as an inventor on several international patents to treat neurodegenerative diseases, including AD and other dementias.

## Publisher's note

All claims expressed in this article are solely those of the authors and do not necessarily represent those of their affiliated organizations, or those of the publisher, the editors and the reviewers. Any product that may be evaluated in this article, or claim that may be made by its manufacturer, is not guaranteed or endorsed by the publisher.

